# Acceptability and deliverability of an auditory rhythmical cueing (ARC) training programme for use at home and outdoors to improve gait and physical activity post-stroke

**DOI:** 10.1186/s40945-021-00126-x

**Published:** 2022-01-04

**Authors:** Patricia McCue, Lisa Shaw, Silvia Del Din, Heather Hunter, Sue Lord, Christopher I. M. Price, Helen Rodgers, Lynn Rochester, Sarah A. Moore

**Affiliations:** 1 Stroke Research Group, Population Health Sciences Institute, Faculty of Medical Sciences, Henry Wellcome Building, The Medical School, Framlington Place, Newcastle University, Newcastle upon Tyne, NE2 4HH UK; 2 Institute of Translational and Clinical Research, Faculty of Medical Sciences, Newcastle University, Newcastle upon Tyne, NE1 7RU UK; 3 Newcastle upon Tyne Hospitals NHS Foundation Trust, Royal Victoria Hospital, Queen Victoria Road, Newcastle upon Tyne, NE1 4LP UK; 4 Auckland University of Technology, 55 Wellesley St E, Auckland, 1010 New Zealand; 5 Stroke Northumbria, Northumbria Healthcare NHS Foundation Trust, Rake Lane, North Shields, Tyne and Wear, NE29 8NH UK; 6 Department of Sport, Exercise, and Rehabilitation, Faculty of Health and Life Sciences, Northumbria University, Newcastle upon Tyne, NE7 7XA UK

**Keywords:** Acceptability, Stroke, Gait, Exercise, Auditory rhythmical cueing

## Abstract

**Background:**

Although laboratory studies demonstrate that training programmes using auditory rhythmical cueing (ARC) may improve gait post-stroke, few studies have evaluated this intervention in the home and outdoors where deployment may be more appropriate. This manuscript reports stakeholder refinement of an ARC gait and balance training programme for use at home and outdoors, and a study which assessed acceptability and deliverability of this programme.

**Methods:**

Programme design and content were refined during stakeholder workshops involving physiotherapists and stroke survivors. A two-group acceptability and deliverability study was then undertaken. Twelve patients post-stroke with a gait related mobility impairment received either the ARC gait and balance training programme or the gait and balance training programme without ARC. Programme provider written notes, participant exercise and fall diaries, adverse event monitoring and feedback questionnaires captured data about deliverability, safety and acceptability of the programmes.

**Results:**

The training programme consisted of 18 sessions (six supervised, 12 self-managed) of exercises and ARC delivered by a low-cost commercially available metronome. All 12 participants completed the six supervised sessions and 10/12 completed the 12 self-managed sessions. Provider and participant session written records and feedback questionnaires confirmed programme deliverability and acceptability.

**Conclusion:**

An ARC gait and balance training programme refined by key stakeholders was feasible to deliver and acceptable to participants and providers.

**Trial registration:**

ISCTRN 12/03/2018.

**Supplementary Information:**

The online version contains supplementary material available at 10.1186/s40945-021-00126-x.

## What’s already known about this topic

Auditory rhythmical cueing improves walking following stroke when delivered in the laboratory or clinical settings. Limited research exists, however, on the use of ARC in the home and outdoors where deployment may be more appropriate.

## What does the study add (one or two sentences)

The study demonstrated that an ARC gait and balance training programme can be delivered in the home and outdoors. The programme was acceptable to both stroke survivors and therapists.

## Background

Although up to 80% of stroke survivors may eventually recover their ability to walk short distances [1], many do not achieve the locomotor capacity necessary for ‘real-world’ walking [2]. Gait impairments can limit household and outdoor ambulation post-stroke [3] and are associated with increased dependency in activities of daily living and reduced quality of life [4]. Typical impairments commonly observed post-stroke include reduced walking speed, decreased stride length/cadence and increased temporal asymmetry [5, 6]. The ability to walk safely and unsupervised around the home and outdoors is fundamental to independent living and as such is an important topic in stroke rehabilitation [7]. Stroke survivors view the ability to walk safely and effectively outdoors as a top priority [8], but unfortunately this is unachievable for many who as a result are confined to home [7, 9].

A potential method of enhancing the efficacy of gait rehabilitation post-stroke is auditory rhythmical cueing (ARC). ARC provides auditory feedback to target gait and physical activity. A metronome beat or music is delivered during exercise training in order to normalise and entrain stepping [10]. The efficacy of ARC has been well established in Parkinson’s disease over the last 20 years [11], and this intervention has more recently been utilised in stroke.

ARC gait training may confer benefits including increased practice of walking which is a recognized key component in recovery post-stroke [10, 12]. A recent systematic review [13] reported significant improvements in gait velocity, cadence and stride length following an ARC intervention compared to control groups receiving other types of rehabilitation. Whilst this suggests promise for ARC as a tool for improving gait, much of this work on ARC in stroke was ward or laboratory based which limits application of findings to ‘real world’ walking. Real world walking requires the ability to change speed and direction, for example, when walking in crowds or across roads, endurance to enable participation in community settings, and the ability to negotiate different terrains during different weather or ambient conditions [14]. Rather than using ARC to target aspects of efficient and effective walking, the studies in the review predominantly targeted laboratory based overground indoor walking in a straight line. The studies included in the review were also limited by size, bias (e.g., only 25% of the studies had blinded outcome assessments) and a large proportion were conducted over 10 years ago.

One recent study has examined the use of ARC within the home for stroke survivors [15]. This small pilot study (*n* = 12) evaluated ARC delivered whilst the stroke survivors stepped on the spot and reported that this programme was feasible, well-tolerated and improved walking ability. Whilst this is promising early data to support the use of ARC in the home, bigger studies and those which include different aspects of walking e.g., turning, and outdoor walking are needed to evaluate this treatment further.

To inform the design of a pilot randomised controlled trial of an ARC gait and balance training programme for use by stroke survivors in the home and outdoors, we undertook the work reported in this manuscript which aimed to refine a prototype ARC programme and then to assess whether the programme was acceptable and deliverable.

## Methods

### Refinement of a prototype ARC programme

Literature on the content and dose of promising previous stroke ARC gait and balance programmes informed the development of a prototype programme_._ [12, 13, 15]. This programme and the related materials were taken to stakeholder workshops to refine content. The participating stakeholders were physiotherapists working in stroke services and stroke survivors. Workshop participants were asked to review the programme and materials for utility and quality of content. Materials included low-cost commercially available metronomes, examples of potential exercise handouts, and a video which showed how to operate a metronome and its use during balance and gait exercises. Verbal discussions were held about the materials with notes taken by a study team attendee, and participants also completed a series of 5-point Likert scale questions (1 ‘strongly disagree’ to 5 ‘strongly agree’) which are shown in the Supplementary Materials Appendix A. At the stakeholder workshops, physiotherapists were also asked to provide additional verbal feedback about aspects of the future acceptability and deliverability study design. The workshops were video recorded.

Following the stakeholder workshops, the video recordings were reviewed with the written notes taken during the sessions and a summary of findings created. Responses to Likert scale questions were collated. Data were used to refine the content of the ARC gait and balance training programme and inform aspects of the acceptability and deliverability study design.

### Acceptability and deliverability study

#### Study design, sample size and setting

A two-group acceptability and deliverability study was conducted. Group one received the developed ARC gait and balance training programme. Group two received the gait and balance training programme but without ARC. This design was chosen to reflect the planned future pilot randomized controlled trial where the gait and balance training programme without ARC would be the control group. The pre-specified sample size was 12 participants and a simple group allocation process was used. The first eight enrolled participants were allocated to group one and the second four participants to group two. The sample size of 12 participants was selected to allow small scale exploration of both programmes before a larger appropriately sized pilot trial. A greater number of participants were allocated to group one because ARC gait and balance training following stroke is relatively novel therefore may be less acceptable/deliverable, whereas gait and balance programmes without ARC are commonly delivered in the home and community in clinical practice. Participants were recruited from two NHS community stroke services in the North East of England. The training programmes were delivered in the participants’ homes and outdoors.

#### Participants

Community dwelling adults within 24 months of stroke (first ever or recurrent) who could walk independently for more than 10 m (with or without a stick) indoors but had a gait-related mobility impairment resulting from their stroke were eligible. Gait-related mobility impairments were based on the routine clinical observation of NHS professionals who identified patients to take part in the study or patient self-report including: e.g. gait asymmetry, reduced walking speed, reduced balance, reduced walking confidence.

Individuals were excluded if they were currently undertaking any active physiotherapy, had other neurological or orthopaedic conditions affecting gait (e.g. Parkinson’s disease, rheumatoid arthritis) or if they had any diagnosis likely to interfere with adherence to training or which predisposed to falls (e.g. uncorrected hearing problems, registered blind). In addition, individuals unlikely to be able to follow study procedures due to cognitive impairment or communication difficulties were also excluded. All participants provided written informed consent. London - City and East Research Ethics Committee granted ethical approval for this study (REF 18/LO/0115, 12th January 2018).

#### Group one: ARC gait and balance training programme

Table 1 provides a summary description of the ARC gait and balance training programme using the Template for Intervention Description and Replication (TIDieR) framework [16].

**Table 1:** Description of the ARC gait and balance training programme using TIDieR framework

TIDieR component	Description
Why (rationale)	In auditory rhythmical cueing gait training, a metronome beat provides auditory feedback during exercise to train stepping. ARC training has been found to improve gait velocity, cadence and stride length in laboratory settings
What (materials):	Metronome: Musedo Metro Tuner MT-100 or Metronome app: ‘ZyMi’ for android or ‘Pro Metronome’ for iOS. Participant exercise manual. Access to exercise videos online: https://youtu.be/INlddw1TugA.
What (procedures)	A total of 10 different home and outdoor gait and balance exercises undertaken with auditory rhythmical cueing.
Who provided	A research physiotherapist with specialist stroke skills and over 20 years clinical experience (**), and a stroke researcher with a background in psychology (**).
How (delivery)	Three exercise sessions per week for 6 weeks. Six sessions were supervised by the providers described above (once per week) and 12 were self-managed sessions (two per week). All outdoor walking sessions were supervised. Telephone support was available if required.
Where	Participants’ homes and outdoors.
When and how much	Eighteen × 30 min sessions (three per week for 6 weeks).
Tailoring	Exercises were gradually progressed according to patient ability by increasing the speed/intensity, duration or amount. Outdoor walking was introduced at week 4.
How well (planned)	Providers were trained and delivery of the entire programme to one participant was reviewed, by the programme lead (** highly specialised stroke physiotherapist and clinical academic). Providers buddied up for the first four participants to observe each other and provide feedback. Providers made written notes about supervised session content. Providers also asked participants to describe and demonstrate exercises undertaken in self management sessions to allow for review and advice as required. Participants were asked to record completion of all sessions in a diary.

The programme consisted of three 30-min training sessions per week for 6 weeks (total 18 sessions) undertaken in home and outdoor settings. This dose and duration was selected based on findings from previous cueing studies in stroke delivered in the laboratory, on the ward and in the home [17], and Parkinson’s disease literature [18].

ARC was provided with either a commercially available metronome (Metro Tuner MT-100 by Musedo) or a free metronome app for a mobile phone: ‘ZyMi’ for android or ‘Pro Metronome’ for iOS. Participant preference led the choice of metronome. The frequency of the auditory cue depended on the type of training and the auditory cue had a regular pattern. A single tone rather than separate tones to cue each leg was used as this approach has been found to be most preferable for stroke patients and is more likely to aid compliance if used as a training tool in rehabilitation [19]. A total of 10 gait and balance exercises were used with ARC. Examples include ‘weight shifting from side-to-side’ and ‘maneuvering between objects’ (documentation shown in Supplementary Materials Appendix B). Exercises were gradually progressed according to the patient’s ability by increasing the speed/intensity, duration or amount.

One training session per week was supervised by a provider from the study team (PM: stroke researcher with a background in psychology or HH: research physiotherapist with stroke specialist skills and over 20 years of clinical experience) and the other two sessions were self-managed. During the supervised sessions, the study provider taught the participant the ARC gait and balance exercises and selected the frequency for the auditory cue during each exercise. During self-managed sessions, participants enacted the exercises that they had been taught in the supervised sessions. Participants were also provided with a paper training manual which included illustrations and descriptions of the exercises to be undertaken. In addition, videos of exercises were available online.

During weeks 4–6, the supervised session focused on walking outdoors. In these sessions, the metronome frequency was initially set at the participant’s self-selected stepping frequency. This is potentially the most effective method of cueing in stroke [20].

Standardising and assessing programme deliverability, and reporting adherence were important aspects of this study and several methods were incorporated into the programme design to address this. The two providers received face-to-face training from the programme lead (highly specialised stroke physiotherapist and clinical academic) in advance of the study start. The study lead also observed each of the providers delivering the programme to one participant to confirm correct delivery. Furthermore, both providers attended all sessions for the first four participants to observe each other and provide feedback about appropriate delivery. During supervised sessions, providers made written notes about session content including exercise enactment, progression and session duration. To check that participants were completing the self-management aspects of the programme as intended, providers asked for a description and demonstration of what had been undertaken and provided participants with feedback as required. Providers also made notes about participant use of study materials including paper exercise instructions and videos, and metronome preferences. Participants were asked to record session completion in a diary section within the issued paper-training manual.

#### Group two: gait and balance training programme without ARC

Participants in group two undertook the gait and balance training programme without ARC. The dose, duration, mode of delivery, exercises, materials (excluding ARC or reference to ARC) and records maintained were identical to group one. At supervised sessions, participants were given basic instructions about exercises but no cues of an auditory nature e.g. any verbal timing cues.

#### Data collection

Participant demography, stroke characteristics, gait performance and other health parameters were recorded on enrolment into the study. These included: sex; age; pre-stroke walking status (with/without stick); pre-stroke disability (Modified Rankin Scale) [21]; stroke type and subtype; time since stroke; current stroke impairment (National Institute of Health Stroke Scale) [22] and disability (Modified Rankin Scale) [21]; walking aid use; ankle foot orthosis use; walking speed (average speed measured over five × 4 m trials with/without stick at self-selected pace, 4 m walk selected because this distance was feasible for measurements in participants homes); current cognitive function (Montreal Cognitive Assessment) [23]; mood (Physical Health Questionnaire-9) [24] and fatigue (Fatigue Assessment Scale), [25].

The following data were collected during or at the end of each participant’s involvement in the study to determine acceptability and deliverability:

1. ARC and/or gait and balance training programme delivery

Data recorded included provider written notes about face-to-face sessions and the participant’s self-completion session diary.

2. Safety including falls

Researchers collected data about any adverse and serious adverse event using standard definitions. To collect data about falls, participants were issued with a study designed falls diary which they were asked to complete applying a standard definition for a fall [26]. Providers of the study training programmes checked that participants were maintaining their falls diary and assisted with completion if required.

3. Participant and provider feedback about ARC and/or gait and balance training

Participants and providers of the ARC and/or gait and balance training programmes completed questionnaires developed for the study. Responses to questions were captured on a 5-point Likert Scale (1 ‘strongly disagree’ to 5 ‘strongly agree’). Participant questions included ease of participating in the study and ARC and/or gait and balance training, and were informed by questions used in a previous feasibility study [27]. Provider questions covered the content of the ARC and/or gait and balance training programme including duration of sessions, exercises and available materials. Providers could also provide additional free text comments about aspects of the programme. Providers completed a separate questionnaire after delivery of the training programme to each individual participant.

#### Data analysis

Quantitative data are reported descriptively. Free text data were examined and summarised.

## Results

### Refinement of a prototype ARC programme

Two stakeholder workshops were undertaken in North East England. Nine physiotherapists working in inpatient and community stroke services attended the first workshop and four stroke survivors attended the second.

Seven commercially available metronomes were discussed and graded by the workshop participants. Two key points emerged from discussion: ease of use and cue delivery. Discussion on ease of use focused on the size of the metronome screen and buttons, potential confusion if there were a number of buttons and practicalities of set up if the stroke survivor could only use one arm. One of the metronomes was positioned in the ear and participants highlighted this may lead to problems for people with hearing aids. Some of the cues delivered by the metronomes were deemed too quiet and the tone of some metronomes were preferred to others. In the physiotherapist workshop the use of a commercially available ARC app was suggested, but some concerns were expressed as to whether stroke survivors could use this technology. This was further explored at the stroke survivor workshop where all participants indicated they would be happy to use an app. Workshop attendees graded the metronomes using seven 5-point Likert Scale questions which gave a maximum score of 35 points per metronome. Stroke survivors also graded the additional ARC app suggested by the physiotherapists. The mean scores for each metronome can be found in Supplementary material Appendix A. The most popular metronome was the ‘Metro Tuner MT-100’.

A total of three prototype ARC gait and balance exercise participant handouts were discussed and graded by the workshop participants. The main point emerging from both the stroke survivor and the physiotherapist workshops was the need for additional detail to be included on the handouts to assist participant understanding. In particular, the cueing tempo during set exercises needed to be more clearly outlined and written instructions on exercise progressions and technique needed more detail and clearer explanation. Participants thought the pictures provided to supplement the text in the handouts worked well. In terms of grading, six 5-point Likert scale questions were completed (Supplementary material Appendix A). Overall, the majority of the responses were positive.

The video demonstrating how to operate a metronome and examples of using it during balance and gait exercises was also discussed and graded. Overall, participants thought the demonstrations in the video were easy to follow and an effective way of reinforcing the handouts and providing guidance and motivation. Participants valued that the video showed a stroke survivor undertaking the exercises in home and outdoor settings. Participants highlighted that it would be useful to have two sets of videos to demonstrate how the exercises should be undertaken with and without a walking aid, including stroke survivors with different levels of ability. Some concerns were raised by the physiotherapist group that some stroke survivors may struggle/not be able to access the video technology. These concerns were not echoed by the stroke survivors. Participant rating scores for the video are shown in Supplementary materials Appendix A. The majority of responses were either ‘agree’ or ‘strongly agree’.

Additional feedback from physiotherapists indicated general support for the study plans. Issues discussed included how different types of walking aids, on-going physiotherapy and visual problems may impact on the training programme.

Stakeholder workshop findings informed the design of the training programme and its materials and some aspects of the acceptability and deliverability study e.g. eligibility criteria.

### Acceptability and deliverability study

#### Participant enrolment and characteristics

Twelve participants were enrolled in the study between April and August 2018. Demography, stroke, gait performance and other health characteristics are shown in Table 2.

**Table 2:** Participant characteristics at study enrolment

Characteristic	***n*** = 12
**Sex: n (%)**	
Male	5 (42)
Female	7 (58)
**Age (years)** Mean (SD)	70 (11)
**Pre-stroke walking status: n (%)**	
With stick	1 (8)
Without stick	11 (92)
**Pre-stroke modified Rankin Scale: n (%)**	
0	11 (92)
3	1 (8)
**Cerebral hemisphere affected by stroke: n (%)**	
Right	6 (50)
Left	5 (42)
Bilateral	1 (8)
**Stroke type: n (%)**	
Ischaemic	6 (50)
Intracerebral haemorrhage	4 (33)
Subarachnoid haemorrhage	0
Unable to verify stroke type	2 (17)
**Stroke subtype: n (%)**	
Total Anterior Circulation Stroke	1 (8)
Partial Anterior Circulation Stroke	2 (17)
Lacunar Stroke	3 (25)
Posterior circulation stroke	1 (8)
Unable to verify stroke subtype	5 (42)
**Time from stroke (months)** Mean, (SD), [range]	13, (5.6), [6–23]
**National Institutes of Health Stroke Scale** Mean, (SD), [range]	2.8, (1.), [1–6]
**Modified Rankin Scale: n (%)**	
0	3 (25)
1	2 (17)
2	1 (8)
3	6 (50)
**Walking aid use: n (%)**	2 (17)
**Ankle foot orthosis use: n (%)**	4 (34)
**Walking speed (metres per second)** Mean, (SD), [range]	0.71, (0.33), [0.20–1.25]
**Montreal Cognitive Assessment Score** Mean, (SD), [range]	24, (3), [19–29]
**Physical Health Questionnaire − 9** Mean, (SD), [range]	6.3, (7.4), [0–24]
**Fatigue Assessment Scale score** Mean, (SD), [range]	23.5, (10.3), [10–40]

#### ARC and/or gait and balance training programme delivery

All twelve participants completed the six supervised training sessions. For the unsupervised sessions (12 in total), ten participants reported completing all 12 and two reported completing 10/12. Provider observation of exercise enactment during the supervised sessions indicated that participants were correctly undertaking the exercises during unsupervised sessions. Providers were able to progress exercises as per the programme guidance with the individual participants. Most participants used the paper-based materials to guide exercise practice with only two participants reporting use of the training videos. Participants reported finding the exercise and falls diaries easy to complete. Thirty minutes was adequate for completion of intended content at supervised sessions with the exception of those sessions which involved outdoor walking. For these sessions, providers reported that more than 30 min was required.

For the eight participants who completed the ARC training, all chose to use the ‘metro tuner’ metronome rather than an app and none of the metronomes malfunctioned or ran out of battery charge during the study period. Participants reported that they felt confident and safe using the metronome alone in the self-managed sessions. Training providers observed that all participants were able to time their footfalls to the metronome auditory cues.

#### Safety including falls

One participant from Group one suffered one serious adverse event during the study timeframe. The participant was shopping and a fall occurred on an escalator which resulted in a fractured neck of femur, and hospitalisation. This event occurred after the end of the participant’s ARC gait and balance training programme. One other participant in Group one fell twice during the programme delivery time period. Both of these falls were minor indoor trips that did not lead to injury and did not occur whilst undertaking the ARC gait and balance training programme.

#### Participant and provider feedback about ARC and/or gait and balance training

Participant and provider feedback responses are shown in Tables 3 and 4 respectively. Responses from participants were mainly positive and all would recommend the training to other people. One participant was unsure about the level of information provided.

**Table 3:** Participant feedback about the ARC and/or gait and balance training programme

Feedback question	Responses % per question					
	Strongly disagree	Disagree	Unsure	Agree	Strongly agree	Participants n=
1. I found the exercise sheets/videos easy to follow during the unsupervised sessions	0	0	0	73	27	11^b^
2. I found it easy to do the exercises to the beat of the metronome ^a^	0	0	0	86	14	7
3. I had enough information to do the exercises without the therapist	0	0	9	45.5	45.5	11
4. It was helpful in improving the way that I walk	0	0	9	55	36	11
5. It built confidence in overcoming barriers related to walking	0	0	0	27	73	11
6. I felt safe doing the exercise programme	0	0	0	18	82	11
7. I would recommend the exercise programme to other people who have problems with walking after stroke	0	0	0	0	100	11

^a^Question 2 only applied to the ARC gait and balance training group. ^b^One of the 12 participants did not complete the questionnaire as they were hospitalised due to a serious adverse event

**Table 4:** Provider feedback about the ARC and/or gait and balance training programme for each participant

Feedback question	Responses % per question					
	Strongly disagree	Disagree	Unsure	Agree	Strongly agree	n=
1. Length of face-to-face sessions of 30 min was adequate to teach the protocol	0	0	0	50	50	12
2. 18 × 30 min sessions were an appropriate length for participant to target their gait and balance	0	0	17	8	75	12
3. The intervention exercises and progressions were appropriate for the participant	0	0	0	8	92	12
4. I found the handbook and falls diary effective for informing the participant about the intervention	0	0	0	0	100	12
5. I feel the videos were effective for informing the participant about the intervention	0	0	66	17	17	12
6. I feel that the combination of face-to-face and self-managed sessions were adequate to administer the intervention properly	0	0	0	0	100	12
7. I feel the telephone support sessions adequate for needs of the participant	0	0	33	0	67	12
8. The home setting of the sessions was appropriate for intervention delivery	0	0	0	0	100	12

Responses from providers were also mainly positive although there were mixed views about the video material and telephone support. Free text comments from providers reported that a session duration of 30 min was not adequate for outdoor walking training. In addition, it was noted that two exercises were very similar (180 degree and 360 degree turns) and would likely be better merged into one.

## Discussion

This work has demonstrated that an ARC gait and balance training programme informed by key stakeholder input is deliverable in the home and outdoors, and is acceptable to both patients and providers. Stakeholder workshops involving physiotherapists and stroke survivors were important for refining training programme components which subsequently enabled creation of a clear set of materials to guide participation. The acceptability and deliverability study demonstrated that stroke survivors were able to undertake the programme, use the materials and perceived it to be of benefit.

A review of previous literature on ARC gait and balance programmes informed the prototype ARC intervention which stroke survivors and physiotherapists then helped to refine. It is important that intervention design incorporates the voices of patients and the public from conception to dissemination, implementation and impact [28]. User input has been shown to increase the probability of a successful design and this approach has been used effectively within stroke studies [29]. In our study, user involvement assisted with the selection of an appropriate metronome and resulted in iterative development of handouts and videos to improve content. In addition, comments suggested the need to design a separate set of materials for people using a walking stick. Physiotherapists also influenced eligibility criteria for the acceptability and deliverability study.

During the acceptability and deliverability study, providers and the study lead observed correct participant enactment of the exercises during the supervised sessions and for those participants undertaking ARC, correct use of the metronome. This indicated that provider instruction and handbooks/video were adequate to guide the training programme, and participants were able to undertaken the content as anticipated. It was encouraging to find that the relatively low-cost commercially available metronome was well tolerated and easy to use. All participants were able to time their footfall to the metronome cues despite a range of functional deficits. The use of this low-cost technology supports a recent call for the use of affordable technology within healthcare in the NHS Long-Term Plan [30].

The training programme combined supervised and self-managed sessions. The rationale for this approach was to increase the amount of training, as evidence supports higher doses of rehabilitation training lead to better outcomes post-stroke [31], without the cost of face-to-face supervision. This mode of delivery also aimed to increase self-efficacy through self-management as has been observed in previous self-management stroke rehabilitation interventions [32]. All participants completed all of the supervised sessions and 10/12 completed all of the self-managed sessions indicating that this type of approach was well tolerated.

Participants were asked to record that they had undertaken exercise sessions on a diary included in the paper training manual. Currently there is little evidence to guide how adherence to exercise during rehabilitation should be recorded [33], however, diaries and logbooks are currently most frequently used [34]. Poor completion of exercise diaries has been previously observed [35], but this did not appear to be the case in our study where completion was adequate. This may have been because programme providers regularly reviewed the paper diaries and supported completion as needed, and this model would be adopted in future work.

Falls are common after stroke and can lead to long-term disability and reduced quality of life [36]. Three falls were recorded as part of the acceptability and deliverability study, occurring outside of times when participants were undertaking the gait and balance training programme. Whilst these falls were not considered a direct study safety issue, the training programme may have led to increased confidence in walking and an increase in daily physical activity, predisposing to falls outside the training programme. This important area will be further explored in the future pilot randomised controlled trial.

As well as assessing delivery and safety of the training programme, participant and provider views were captured using a simple questionnaire. Participants predominantly reported that they found the programme easy to follow, felt safe undertaking the exercises and found them helpful for their walking. All of the participants would recommend the programme to other stroke survivors. With walking problems experienced by 80% of stroke survivors [37] and the need for further research on walking interventions highlighted within the top ten priorities for stroke research [8], the views about this programme support its further evaluation.

Providers were also positive about the training programme only suggesting some minor adaptations e.g. more time to deliver outdoor walking sessions and minor adjustments to some exercises. The two providers however were members of the study team, which could have biased their views on the programme. Although NHS healthcare professionals inputted into the design of the programme, as they were not involved in delivery in this study it was not possible to get further views. Exploring wider healthcare professional views in the future would be beneficial.

Providers were unsure about the use of the videos but this was due to the fact the videos were not used by the majority of participants. In developing the programme, it was felt that having both paper based and video resources demonstrating the exercises may aid adherence and the use of video was in keeping with suggestions that technology should be utilised to enhance exercise adherence. It is interesting, therefore, that many stroke survivors opted for the more standard paper-based tools which have been shown to be equally effective as technological alternatives [38].

## Conclusion

This work has demonstrated that an ARC gait and balance training programme designed for use in the home and outdoors can be delivered and is acceptable to both patients and providers. It was feasible to use a low-cost commercially available metronome to deliver the ARC and paper-based exercise materials. A pilot randomised controlled trial using the programme is on-going [39].

## Supplementary Information

**Additional file 1.** Supplementary materials.

## Data Availability

Not applicable. Due to the nature of this study data will not be made available.

## Abbreviations

ARC: Auditory rhythmical cueing
HCPs: Health care professionals
NHS: National health service
TIDieR: Template for intervention description and replication
