# Supplementary material for: Acceptability and deliverability of an auditory rhythmical cueing (ARC) training programme for use at home and outdoors to improve gait and physical activity post-stroke
Source: Arch Physiother. 2022 Jan 4;12:1. doi: 10.1186/s40945-021-00126-x (PMC8725469; doi:10.1186/s40945-021-00126-x)
Supplement: Supplementary file 1 — Additional file 1. Supplementary materials. [file 40945_2021_126_MOESM1_ESM.docx]

***Supplementary materials***

***Appendix A Results from stakeholder workshops***

***Metronome grading by physiotherapists***

| Metronome  model | Metro Tuner | Thomann CN-10 | Quik Tune QT3 | Korg IE1M | Meideal M50 | Pure Tone | Chord CMT-1 |
| --- | --- | --- | --- | --- | --- | --- | --- |
| Mean score for  each device (n=9) | 33 | 27 | 33 | 20 | 27 | 22 | 29 |

***Metronome and ARC app grading by stroke survivors***

| Metronome  model | Metro Tuner | Thomann CN-10 | Quik Tune QT3 | Korg IE1M | Meideal M50 | Pure Tone | Chord CMT-1 | ZyMi App |
| --- | --- | --- | --- | --- | --- | --- | --- | --- |
| Mean score for  each device (n=4) | 33 | 27 | 33 | 20 | 27 | 22 | 29 | 34 |

***Physiotherapist grading for balance exercise 1 handout***

| Question | Response: n(%) | | | | |
| --- | --- | --- | --- | --- | --- |
|  | **Strongly disagree** | **Disagree** | **Undecided** | **Agree** | **Strongly agree** |
| The content was organised and easy to follow | 0 | 0 | 0 | 4 (50) | 4 (50) |
| The instructions were clear | 0 | 0 | 1 (12.5) | 4 (50) | 3 (37.5) |
| The pictures were helpful | 0 | 0 | 0 | 4 (50) | 4 (50) |
| There is enough information for the patient to do the exercises without the therapist | 0 | 0 | 0 | 5 (62.5) | 3 (37.5) |
| It is clear how many times and for how long the patient would have to do the exercises | 0 | 0 | 0 | 7 (87.5) | 1 (12.5) |
| I think the patient would find it clear how to set the metronome up for the exercises | 0 | 0 | 1 (12.5) | 7 (87.5) | 0 |

***Physiotherapist grading for balance exercise 2 handout***

| Question | Response: n(%) | | | | |
| --- | --- | --- | --- | --- | --- |
|  | **Strongly disagree** | **Disagree** | **Undecided** | **Agree** | **Strongly agree** |
| The content was organised and easy to follow | 0 | 0 | 0 | 3 (43) | 4 (57) |
| The instructions were clear | 0 | 0 | 1 (14) | 3 (43) | 3 (43) |
| The pictures were helpful | 0 | 0 | 1 (14) | 2 (29) | 4 (57) |
| There is enough information for the patient to do the exercises without the therapist | 0 | 0 | 0 | 4 (57) | 3 (43) |
| It is clear how many times and for how long the patient would have to do the exercises | 0 | 0 | 0 | 5 (71) | 2 (29) |
| I think the patient would find it clear how to set the metronome up for the exercises | 0 | 0 | 1 (14) | 5 (71) | 1 (14) |

***Physiotherapist grading for walking exercise 1 handout***

| Question | Response: n(%) | | | | |
| --- | --- | --- | --- | --- | --- |
|  | **Strongly disagree** | **Disagree** | **Undecided** | **Agree** | **Strongly agree** |
| The content was organised and easy to follow | 0 | 0 | 0 | 3 (43) | 4 (57) |
| The instructions were clear | 0 | 0 | 2 (29) | 2 (29) | 3 (43) |
| The pictures were helpful | 0 | 0 | 1 (14) | 2 (29) | 4 (57) |
| There is enough information for the patient to do the exercises without the therapist | 0 | 0 | 1 (14) | 3 (43) | 3 (43) |
| It is clear how many times and for how long the patient would have to do the exercises | 0 | 0 | 0 | 5 (71) | 2 (29) |
| I think the patient would find it clear how to set the metronome up for the exercises | 0 | 0 | 1 (14) | 5 (71) | 1 (14) |

***Stroke survivor grading for balance exercise 1 handout***

| Question | Response: n(%) | | | | |
| --- | --- | --- | --- | --- | --- |
|  | **Strongly disagree** | **Disagree** | **Undecided** | **Agree** | **Strongly agree** |
| The content was organised and easy to follow | 0 | 0 | 0 | 0 | 4(100) |
| The instructions were clear | 0 | 0 | 0 | 1 (25) | 3 (75) |
| The pictures were helpful | 0 | 0 | 0 | 0 | 4 (100) |
| There is enough information for the patient to do the exercises without the therapist | 0 | 0 | 0 | 1 (25) | 3 (75) |
| It is clear how many times and for how long the patient would have to do the exercises | 0 | 1 (25) | 0 | 1 (25) | 2 (50) |
| I think the patient would find it clear how to set the metronome up for the exercises | 0 | 0 | 0 | 1 (34) | 2 (66) |

***Stroke survivor grading for balance exercise 2 handout***

| Question | Response: n(%) | | | | |
| --- | --- | --- | --- | --- | --- |
|  | **Strongly disagree** | **Disagree** | **Undecided** | **Agree** | **Strongly agree** |
| The content was organised and easy to follow | 0 | 0 | 0 | 0 | 4 (100) |
| The instructions were clear | 0 | 0 | 0 | 1 (75) | 3 (75) |
| The pictures were helpful | 0 | 0 | 0 | 1 (75) | 3 (75) |
| There is enough information for the patient to do the exercises without the therapist | 0 | 0 | 0 | 1 (75) | 3 (75) |
| It is clear how many times and for how long the patient would have to do the exercises | 0 | 1 (25) | 0 | 0 | 3 (75) |
| I think the patient would find it clear how to set the metronome up for the exercises | 0 | 0 | 0 | 0 | 3 (100) |

***Stroke survivor grading for walking exercise 1 handout***

| Question | Response: n(%) | | | | |
| --- | --- | --- | --- | --- | --- |
|  | **Strongly disagree** | **Disagree** | **Undecided** | **Agree** | **Strongly agree** |
| The content was organised and easy to follow | 0 | 0 | 0 | 0 | 4 (100) |
| The instructions were clear | 0 | 0 | 0 | 1 (25) | 3 (75) |
| The pictures were helpful | 0 | 0 | 0 | 0 | 4(100) |
| There is enough information for the patient to do the exercises without the therapist | 0 | 0 | 0 | 2 (50) | 2 (50) |
| It is clear how many times and for how long the patient would have to do the exercises | 0 | 1 (25) | 0 | 0 | 3 (75) |
| I think the patient would find it clear how to set the metronome up for the exercises | 0 | 0 | 0 | 0 | 3 (100) |

***Physiotherapist grading for video***

|  | Response: n(%) | | | | |
| --- | --- | --- | --- | --- | --- |
|  | **Strongly**  **Disagree** | **Disagree** | **Undecided** | **Agree** | **Strongly**  **Agree** |
| **I would be happy to open this up and demonstrate the process and the exercises to the patient** | 0 | 0 | 0 | 4 (44) | 5 (56) |
| **I feel the patient would be happy to open and play the video from a computer, tablet or phone** | 0 | 0 | 3 (37.5) | 4 (50) | 1 (12.5) |
| **The menu instructions on the video itself are clear and understandable for the patient** | 0 | 0 | 0 | 6 (67) | 3 (33) |
| **The patient would be likely to use this by themselves** | 0 | 0 | 1 (12.5) | 6 (75) | 1 (12.5) |
| **I think this video will be useful for the patient** | 0 | 0 | 0 | 4 (50) | 4 (50) |
| **There is enough information on the video for the patient to do the exercises without the therapist** | 0 | 0 | 0 | 6 (75) | 2 (25) |

***Stroke survivor grading for video***

|  | Response: n(%) | | | | |
| --- | --- | --- | --- | --- | --- |
|  | **Strongly**  **Disagree** | **Disagree** | **Undecided** | **Agree** | **Strongly**  **Agree** |
| **I am happy to open and play the video from a computer, tablet or phone** | 0 | 0 | 0 | 1 (25) | 3 (75) |
| **The menu instructions on the video itself are clear and understandable** | 0 | 0 | 0 | 1 (25) | 3 (75) |
| **I would be likely to use this by myself** | 0 | 0 | 0 | 0 | 4 (100) |
| **I think this video will be useful** | 0 | 0 | 0 | 1 (25) | 3 (75) |
| **There is enough information on the video to do the exercises without the therapist** | 0 | 0 | 0 | 1 (25) | 3 (75) |

**Appendix B** Examples of ARC gait and balance training exercises

| **Exercise** | **Repetitions/time** | **Progressions** |
| --- | --- | --- |
| 1. **Weight shift side to side with ARC device** | 3 x 10 | - Without holding on - Increase the cueing frequency by 5% to work on speed - Reduce the cueing frequency by 5% to work on control - Work on movement selectivity |
| 1. **Weight shift forward and back with ARC device** | 10 with the left foot in front  10 with the right foot in front | - Without holding on - Increase the cueing frequency by 5% to work on speed - Reduce the cueing frequency by 5% to work on control |
| 1. **Stepping forwards and backwards with ARC device** | 10 x forward and back with both feet leading with the right leg  10 x forward and back with both feet leading with the left leg | - Without holding on - Increase the cueing frequency by 5% - Reduce the cueing frequency by 5% to work on control |
| 1. **Side stepping with ARC device** | Continue side stepping for 2 minutes | - Without holding on - Increase the cueing frequency by 5% - Reduce the cueing frequency by 5% to work on control |
| 1. **Turning 180 degrees in both directions with ARC device** | 5 x in one direction  5 x in the other direction | - Increase the cueing frequency by 5% - Reduce the cueing frequency by 5% to work on control - Increase the number of repetitions - Work on the quality of the movement pattern Balance training Walking |
| 1. **Turning 360 degrees with ARC device** | 3 x in one direction  3 x in other direction | - Increase the cueing frequency by 5% - Reduce the cueing frequency by 5% to work on control - Increase the number of repetitions |
| 1. **Forward stepping onto a step with ARC device** | 10 x forward and back with both feet leading with the right leg  10 x forward and back with both feet leading with the left leg | - Increase the cueing frequency by 5% - Reduce the cueing frequency by 5% to work on control - Increase the number of repetitions |
| 1. **Side stepping onto a step with ARC device** | 10 x stepping both feet onto the step and onto the other side and returning to start position | - Increase the cueing frequency by 5% - Reduce the cueing frequency by 5% to work on control - Increase the number of repetitions |
| **Gait training** |  |  |
| 1. **Standing march with ARC device** | 1 minute | - Increase time - Increase the cueing frequency by 5% - Reduce the cueing frequency by 5% to work on control |
| 1. **Walking and turning with ARC device** | 10 short walks | - Increase time - Increase the cueing frequency by 5% - Reduce the cueing frequency by 5% to work on control - Walking and turning - Walking backwards - Walking whilst carrying an object e.g. cup |
| 1. **Maneuvering between objects with ARC device** | Repeat each circuit 5 times (e.g. manoeuvring between two chairs and returning to start position) | - Increase number of circuits - Increase the cueing frequency by 5% - Reduce the cueing frequency by 5% to work on control - Change the objects to make them more difficult to maneuver around |
| 1. **Basic outdoor walking with ARC device** | 5 minutes | - Increase time/distance walked - Increase the cueing frequency - Up and down curbs |
| 1. **Advanced outdoor walking with ARC device** | 5 minutes | - Walking in busy areas e.g. shopping mall - Walking on different surfaces e.g. grass/sand - Up and down hills |

Example of participant exercise instructions

**Balance Training Exercises - Instructions**

**Exercise 1: Weight Shift Side to Side**

**What you will need to do:**

1. Stand up tall with your feet hip-width apart holding onto a surface for support to one side (eg kitchen bench).

2. Start the metronome and listen for the beat.

3. Transfer your weight onto your left leg (keeping both feet on the floor).

4. Transfer your weight onto your right leg.

5. Repeat.

Date: ………………… Beats per minute: ……...………Do 3 sets of 10
